# Talent environment development in Canadian female youth ice hockey: a first look into the Quebec developmental landscape

**DOI:** 10.3389/fpsyg.2026.1829415

**Published:** 2026-06-10

**Authors:** Vincent Huard Pelletier, Ève-Amélie Roy, W. Simard, Joanie Desgagné

**Affiliations:** 1 Hockey Research Laboratory at UQTR, Department of Human Kinetics, UQAC, Saguenay, QC, Canada; 2 Department of Education, UQAC, Saguenay, QC, Canada

**Keywords:** coaching, female, ice hockey, talent development, youth sports

## Abstract

**Introduction:**

In the Canadian context, ice hockey functions as a fundamental agent of socialization and a catalyst for national identity. Despite the exponential rise of female participation—bolstered by the professionalization of the Professional Women’s Hockey League—a significant “gender gap” persists in sports science literature, which traditionally prioritizes male cohorts in talent development research. This investigation pursued three primary objectives: (1) to evaluate the structural validity and internal consistency of the Talent Development Environment Questionnaire-5 (TDEQ-5) within the Quebec female youth hockey context; (2) to examine variances in environmental perceptions based on competitive calibre (A, AA, AAA) and age category (U11–U18); and (3) to quantify the predictive contribution of TDEQ-5 dimensions in shaping athletes’ long-term engagement intentions.

**Methods:**

A sample of 473 female ice hockey players (N = 473) was recruited during a centralized provincial tournament.

**Results:**

Confirmatory factor analyses supported the five-factor structure of the TDEQ-5, although the Alignment of Expectations (AE) dimension exhibited lower internal consistency. MANOVA revealed no significant differences based on competitive caliber, suggesting a structural homogeneity of developmental standards. However, a significant multivariate effect for age was observed, characterized by an erosion of the Support Network (SN) and Alignment of Expectation (AE) among older players (U15 and U18). Regression analysis indicated that TDEQ-5 dimensions accounted for 29.1% of the variance in long-term engagement intentions, with Long-Term Development (LTD) and AE emerging as the most significant predictors.

**Discussion:**

These findings highlight the critical importance of maintaining a supportive climate and ensuring clarity of pathways during the transition to adulthood to foster player retention.

## 1 Introduction

In Canada, ice hockey transcends competition to catalyze national identity and social cohesion. A “cultural totem” (Gruneau and Whitson, 1993), it bridges geographic and linguistic divides as a primary socialization agent for youth (Chamberlain et al., 2021). Beyond identity, participation in organized hockey yields biopsychosocial benefits consistent with those observed in organized youth sports more broadly. Physiologically, it develops complex motor patterns (Jaakkola et al., 2017), aerobic capacity (Ferland et al., 2021), and bone density (Sandström et al., 2000). Psychologically, high-stakes competitive environments necessitate constant emotional regulation (Ansell and Spencer, 2022) and resilience (Davis, 2024), while socially, the hockey milieu promotes life skills such as collective efficacy and interpersonal accountability (Bean et al., 2022). However, youth sport is not inherently positive; if poorly managed, these environments can become catalysts for maladaptive outcomes (Veillette et al., 2025). Excessive psychological pressure, social exclusion, and burnout represent significant risks when the developmental climate fails to align with the athlete’s needs, ultimately leading to premature dropout (Gustafsson et al., 2011; Hill, 2013). Recognizing that sport can promote both positive and negative adaptations underscores the importance of evaluating the specific features of the developmental environment that foster growth or precipitate athletic distress.

A persistent conceptual tension exists between the constructs of talent identification (TID), selection, and development (TD) (Zhao et al., 2024). Traditionally, sports organizations have prioritized TID, focusing on cross-sectional assessments of physiological, anthropometric (Barraclough et al., 2022), and historically psychological characteristics (Abbott and Collins, 2004). This approach is frequently confounded by maturational biases and the relative age effect, leading to the premature exclusion of potential talent (Barnsley, 2025; Huard Pelletier and Lemoyne, 2022). Conversely, TD is increasingly guided by the Holistic Ecological Approach (HEA), which adopts a longitudinal perspective centered on a “nurturing milieu” (Valtiner, 2023). Unlike traditional atomistic models that isolate individual traits, the HEA shifts the analysis to the environment as a whole, examining how interconnected micro-systems (e.g., coaches, peers, family) and macro-systems (e.g., organizational culture) synchronize to support the athlete’s journey (Henriksen et al., 2010; Henriksen and Stambulova, 2017). The quality of this Talent Development Environment (TDE) is a critical determinant in preventing adolescent dropout (Hauser et al., 2024); however, if poorly managed, these environments can also become catalysts for maladaptive outcomes (Veillette et al., 2025). Indeed, attrition often results from environments that fail to meet athletes’ needs (Zhang et al., 2024) or neglect their biopsychological maturity (Dunn et al., 2025). To operationalize this ecological perspective, Martindale et al. (2010) developed the TDEQ, later refined as the TDEQ-5 (Li et al., 2015). High TDEQ scores—notably in Holistic Quality Preparation (HQP)—are strongly correlated with elite status attainment (Li et al., 2018; Martindale et al., 2013). Beyond performance, the framework aligns with Positive Youth Development (PYD), as long-term strategies and clear communication facilitate the transfer of life skills (Holt et al., 2017). In contrast, a lack of Alignment of Expectations (AE) can jeopardize an athlete’s well-being, particularly during precarious adolescent transitions into high-performance tiers (Ivarsson et al., 2015).

Despite these theoretical advancements, a substantial gender gap persists in the sport science literature. Indeed, the vast majority of TDEQ studies are conducted on male cohorts. This is problematic, as significant sex-related differences exist in athletes’ developmental pathways (Bowes and Kitching, 2020), especially in ice hockey (Huard Pelletier et al., 2025). Emerging evidence suggests that women ice hockey players perceive their developmental environment more negatively than men and do not place as much importance on becoming professionals (Bergström et al., 2026). This difference is more prominent in ice hockey than in other team sports (Mehus et al., 2025). Within the Quebec hockey ecosystem—a centralized structure with distinct regional and competitive tiers—no empirical data currently exist regarding whether these environments are optimized for female-specific developmental needs. The Quebec female ice hockey development pathway is characterized by a stratified organizational structure—divided into A, AA, and AAA calibres—differentiated by team density, coaching standards, and institutional support. The ‘A’ calibre represents the grassroots competitive tier, comprising approximately 150 teams province-wide; at this level, development is facilitated by local associations utilizing municipal arenas and volunteer coaches certified at the NCCP (National Coaching Certification Program) Coach 1 or 2 levels (Hockey Québec, 2023). Progression into the ‘Excellence’ stream (AA and AAA) introduces significant structural shifts. The AAA tier, managed under the *Ligue de Hockey d’Excellence du Québec* (LHEQ), is highly centralized and limited to approximately 8 teams per age division to ensure a high concentration of elite talent (LHEQ, 2024). Coaching requirements escalate across these tiers, with AAA programs mandating *High Performance 1* (HP1) certification for head coaches and emphasizing advanced tactical and pedagogical expertise (Hockey Québec, 2023). Furthermore, while ‘A’ and ‘AA’ levels rely on standard community infrastructure, ‘AAA’ athletes are typically integrated into formal *Sports-Études* (sport-study) programs. These programs provide enhanced developmental resources, including daily ice access during school hours, integrated video analysis, and specialized physical preparation facilities (LHEQ, 2024; Ministère de l’Éducation, 2022).

Several concerns have recently emerged among the public regarding the development environment in Quebec’s hockey (Leblanc, 2026). Key issues include high rates of early specialization, a strong relative age effect (Hancock et al., 2013; Huard Pelletier and Lemoyne, 2022), an exodus of elite players to American colleges (Mac, 2022), and historical disparities in resource allocation compared to male counterparts (Hockey Québec, 2021). Furthermore, dropout rates remain critical, with more than 25% of players (both male and female) withdrawing from the sport between the ages of 10 and 15 (Hockey Québec, 2023), often driven by complex psychosocial factors (Werner, 2021). Despite these systemic challenges, current research has yet to differentiate environmental perceptions based on players’ age and caliber. Consequently, it remains unknown which specific dimensions of the environment most influence athletes’ overall perception and their willingness to continue developing within the system.

In this context, the exponential rise of female ice hockey—bolstered by its Olympic permanence and the professionalization of the Professional Women’s Hockey League (2025)—necessitates a critical investigation into the quality of the environments tasked with nurturing this burgeoning talent pool. This study is structured around three objectives: (1) to evaluate the structural validity and internal consistency of the TDEQ-5 within the sociocultural and linguistic context of Quebec female youth hockey; (2) to examine variations in Long-Term Engagement Intentions (LTEI) based on competitive calibre (A, AA, AAA) and age category (U11 through U18); and (3) to quantify the relative predictive contribution of the five TDEQ-5 dimensions in shaping athletes’ LTEI within their current environment.

## 2 Methodology

### 2.1 Ethical considerations and recruitment

This study was conducted in strict accordance with the ethical standards for research involving human subjects and received formal approval from the Institutional Research Ethics Board (2025–2017) of the Université du Québec à Chicoutimi (UQAC). Given the inclusion of youth athletes, a dual-consent procedure was implemented: written parental consent was required for participants under the age of 14, alongside the athlete’s individual assent. Participants aged 14 and older provided independent informed consent in accordance with the Quebec’s Civil Code.

Data collection occurred during a major end-of-season provincial tournament, ensuring representation from all 17 administrative regions of Quebec. The event featured over 120 teams (~2,000 players), representing approximately 25% of the province’s female youth hockey population (Hockey Québec, 2025). As participation was contingent upon finishing first in their respective regions, this centralized purposive sampling strategy targeted a high-performing subset of the provincial ecosystem. This approach provided a comprehensive cross-section of successful developmental environments across all competitive calibres and age categories.

### 2.2 Participants

The final sample comprised 473 female ice hockey players after excluding 49 incomplete profiles (9.4%). Participants were distributed across the primary stages of the developmental pathway: U11 (18.5%), U13 (22.4%), U15 (37.5%), and U18 (18.1%). Regarding competitive levels, the sample included athletes from the ‘A’ (25.3%), ‘AA’ (60.6%), and ‘AAA’ (13.9%) calibers. This distribution reflects a purposive sampling strategy focused on the excellence stream (74.5% representing AA and AAA), which is theoretically consistent with the TDEQ-5’s intended use for evaluating high-performance environments (Martindale et al., 2010).

### 2.3 Measures

*Talent development environment*: the quality of the environment was assessed using the *Talent Development Environment Questionnaire* (TDEQ-5; Martindale et al., 2010). This 25-item instrument measures five primary dimensions: (a) Holistic Quality Preparation (HQP), (b) Long-Term Development (LTD), (c) Support Network (SN), (d) Communication (COM), and (e) Alignment of Expectations (AE). Responses were recorded on a 6-point Likert scale ranging from 1 (*Strongly Disagree*) to 6 (*Strongly Agree*).

*Long-Term Engagement Intention (LTEI)*: To represent a proximal outcome of the TDE, a 3-item scale was developed to assess athletes’ adherence to the female-specific pathway and their intent to persist in their current environment. Sample items include “*I intend to continue playing hockey in my current environment next year*”, “*I am committed to pursuing my development within this program*” and “*I want to continue playing in the same league in the coming seasons*”. Psychometric evaluation confirmed the scale’s unidimensionality (PCA loadings: 0.68, 0.71, 0.84) and acceptable internal consistency (*α* = 0.68) for an exploratory measure (Tabachnick and Fidell, 2019). The LTEI reflects the athletes’ psychological commitment to their specific developmental trajectory, rated on the same 6-point Likert scale.

### 2.4 Data analysis plan

Statistical analyses were performed using Python (v3.10) and the *Pingouin* and *Statsmodels* libraries, with the significance level set at *p* < 0.05. Data were initially screened to identify missing values and multivariate outliers. Distributional assumptions were verified using skewness and kurtosis indices to ensure the data met the normality requirements for parametric testing. Multivariate normality was further assessed using Mahalanobis (Ghorbani, 2019) distance to identify and exclude critical outliers. Descriptive statistics, including means and standard deviations, were computed for each environmental dimension and the LTEI scale. These analyses provided a preliminary overview of participants’ perceptions and allowed for the assessment of score dispersion within the sample.

*Objective 1: Psychometric Validation:* The internal structural validity of the TDEQ-5 within the Quebec female hockey context was evaluated using a Confirmatory Factor Analysis (CFA). Model fit was assessed using well-known indices (Yaşlıoğlu and Yaşlıoğlu, 2020): the Comparative Fit Index (CFI > 0.90), the Root Mean Square Error of Approximation (RMSEA < 0.08), and the Standardized Root Mean Square Residual (SRMR < 0.08). Internal consistency for each sub-dimension was determined using Cronbach’s alpha and McDonald’s omega (Madadizadeh and Bahariniya, 2025) to ensure reliability within this specific population.

*Phase 2: Comparative Analysis (Objective 2):* To determine if environmental perceptions vary across competitive levels (A, AA, AAA) and age categories (U11 to U18), a Multivariate Analysis of Variance (MANOVA) was conducted. This approach was selected to account for the inter-correlations between the five TDEQ dimensions while protecting against Type I error inflation. Significant multivariate effects were followed by univariate ANOVAs and Tukey HSD *post-hoc* tests (Abdi and Williams, 2010) to identify specific group differences.

*Phase 3: Predictive Modeling (Objective 3):* To evaluate the unique contribution of the talent development environment while accounting for demographic and competitive variations, a multiple regression was conducted with age categories and competitive calibers entered as control variables. The five sub-dimensions were entered as independent variables, with the LTEI scale as the dependent variable. To incorporate categorical predictors into the multiple regression model, age categories and competitive calibers were dummy-coded using k-1 variables. The U11 age category and the ‘A’ caliber were designated as the reference categories, ensuring that all reported coefficients reflect specific contrasts against these baseline developmental stages and levels. Prior to analysis, assumptions of linearity, homoscedasticity, and the absence of multicollinearity (Variance Inflation Factor < 5) were verified. This phase aims to quantify the relative contribution of each environmental factor in shaping the overall athlete experience.

## 3 Results

### 3.1 Preliminary analyses and data screening

#### 3.1.1 Data cleaning and distributional assumptions

Univariate normality was assessed for each of the 25 items and the LTEI scale. Absolute values for skewness ranged from 0.08 to 0.55, and kurtosis values ranged from 0.05 to 0.41. All indicators fell well within the recommended thresholds of 2.0 for skewness and 7.0 for kurtosis (Tabachnick and Fidell, 2019), suggesting that the data were sufficiently normal for the planned parametric analyses. Multivariate normality was further examined using Mahalanobis distance; no critical multivariate outliers were identified at the *p* < 0.001 or *p* < 0.05 levels.

#### 3.1.2 Descriptive statistics of environmental dimensions

Table 1 presents the descriptive statistics, including means, standard deviations, and distributional indices for the five TDEQ-5 sub-dimensions and the LTEI.

**Table 1:** Mean, standard deviation and comparison for TDEQ-5 dimensions and LTEI.

Age category	*n*	LTD	HQP	SN	COM	AE	LTEI
U11	90	4.85 (0.86)ᵃ	4.82 (0.86)ᵃ	4.58 (1.25)ᵃ	4.34 (1.00)ᵃ	4.81 (0.96)ᵃ	5.43 (0.81)ᵃ
U13	106	4.87 (0.65)ᵃ	4.64 (0.87)ᵃᵇ	4.46 (1.22)ᵃ	4.37 (0.92)ᵃ	4.75 (0.91)ᵃ	5.39 (0.79)ᵃ
U15	177	4.61 (0.80)ᵃᵇ	4.48 (1.10)ᵇᶜ	3.73 (1.32)ᵇ	4.18 (1.02)ᵃ	4.50 (1.45)ᵃ	4.94 (1.01)ᵇ
U18	100	4.44 (1.31)ᵇ	4.35 (1.41)ᶜ	4.10 (1.54)ᵃᵇ	4.14 (1.39)ᵃ	4.33 (1.47)ᵃ	4.81 (1.23)ᵇ
Total Sample	473	4.67 (0.91)	4.55 (1.09)	4.11 (1.38)	4.24 (1.08)	4.58 (1.27)	4.94 (1.02)

Values are presented as Mean (Standard Deviation). Means in the same column sharing a common superscript letter (a, b, c) are not significantly different (*p* > 0.05) according to Tukey HSD *post-hoc* tests. LTD, Long-Term Development; HQP, Holistic Quality Preparation; SN, Support Network; COM, Communication; AE, Alignment of Expectations; LTEI, Long-Term Engagement Intentions.

The descriptive results indicate that the athletes generally perceived their environment positively, with LTD (*M* = 4.77, *SD* = 0.80) receiving the highest ratings, followed by HQP (*M* = 3.89, *SD* = 0.83). COM (*M* = 3.74, *SD* = 0.93) and SN (*M* = 3.68, *SD* = 1.10) had relatively high scores. Conversely, the AE dimension (*M* = 3.22, *SD* = 0.92) showed the lowest mean score, suggesting a potential area of systemic tension in the Quebec female hockey pathway. The LTEI score (*M* = 4.10) suggests a high level of overall satisfaction with the developmental milieu, providing a solid baseline for subsequent predictive modeling and analysis across calibers and categories.

### 3.2 Phase 1: structural validity and confirmatory factor analysis (CFA)

To address the first objective of this study, a Confirmatory Factor Analysis (CFA) was conducted to test the structural integrity of the original five-factor TDEQ-5 model (Martindale et al., 2010) within the Quebec female ice hockey population. The measured model showed that the 25 items load onto their respective latent factors: Holistic Quality Preparation (HQP), Long-Term Development (LTD), Support Network (SN), Communication (COM), and Alignment of Expectations (AE).

#### 3.2.1 Model fit

Parameters were estimated using the Maximum Likelihood (ML) method and can be seen in Table 2. The global fit of the model was evaluated through a combination of absolute and incremental fit indices. While the chi-square test was significant, *χ*^2^ (265) = 742.31, *p* < 0.001, this was anticipated given the index’s sensitivity to sample size (*N =* 473). Other fit indices indicated a satisfactory to good fit of the theoretical model to the observed data. The *χ*^2^/df ratio of 2.80 below the critical threshold of 3.0, and the incremental fit indices—CFI and TLI—exceeded the 0.90 threshold recommended for robust structural validation.

**Table 2:** Fit indices for the TDEQ-5 five-factor model.

Fit index	Value	Acceptability threshold	Interpretation
*χ*^2^/df	2.80	< 3.0	Satisfactory
CFI	0.918	> 0.90	Satisfactory
TLI	0.904	> 0.90	Satisfactory
RMSEA [90% CI]	0.062 [0.057, 0.067]	< 0.08	Satisfactory
SRMR	0.059	< 0.08	Satisfactory

*χ*^2^/df, normed chi-square; CFI, Comparative Fit Index; TLI, Tucker-Lewis Index; RMSEA, Root Mean Square Error of Approximation; CI, Confidence Interval; SRMR, Standardized Root Mean Square Residual.

#### 3.2.2 Factor loadings and internal consistency

Examination of the standardized factor loadings revealed that all items loaded significantly onto their respective factors (*p* < 0.001), with coefficients ranging from 0.44 to 0.82. These results imply that the items adequately represent the latent constructs they are intended to measure in the context of female hockey. The internal consistency of each dimension was evaluated using Cronbach’s alpha (*α*) and McDonald’s omega (*ω*). As detailed in Table 3, four of the five dimensions demonstrated strong internal consistency. The Alignment of Expectations (AE) dimension exhibited more modest reliability (*α* = 0.56, *ω* = 0.58), an observation consistent with previous international validations of the TDEQ-5 (Li et al., 2015; Thomas et al., 2020), suggesting greater heterogeneity in how developing athletes perceive this specific dimension. The issue stems primarily from item 9, “I am involved in most decisions about my sport development” which has a factor loading of only 0.44. Without this item, the scale’s internal consistency increases from 0.56 to 0.63. To remain true to the questionnaire, it was decided to keep the item. Overall, these results support the structural validity and acceptable reliability of the TDEQ-5 within the Quebec female ice hockey context.

**Table 3:** Average factor loadings and reliability indices for TDEQ-5 dimensions.

Dimension	No. of items	Mean λ	*α*	*ω*
Holistic Quality Preparation (HQP)	7	0.68	0.84	0.85
Long-Term Development (LTD)	5	0.71	0.81	0.82
Support Network (SN)	4	0.65	0.78	0.79
Communication (COM)	4	0.64	0.77	0.77
Alignment of Expectations (AE)	5	0.49	0.56	0.58

### 3.3 Phase 2: comparative analysis across competitive levels and age categories

To address the second objective, a two-way Multivariate Analysis of Variance (MANOVA) was performed to examine potential differences in environmental perceptions based on competitive calibre (A, AA, AAA) and age category (U11, U13, U15, U18).

#### 3.3.1 Multivariate results

The MANOVA revealed a significant multivariate main effect for age category, *λ* = 0.887, *F*(15, 1,173) = 3.47, *p* < 0.001, $\eta_{p}^{2}$ = 0.040. No significant multivariate main effect was observed for competitive caliber; Wilks’ λ = 0.985, *F*(10, 850) = 0.66, *p* = 0.765, $\eta_{p}^{2}$ = 0.008. The interaction effect between calibre and age category was also not statistically significant, Wilks’ λ = 0.941, *F*(30, 1845) = 0.87, *p* = 0.66, $\eta_{p}^{2}$ = 0.014, suggesting that the influence of competitive level on environmental perceptions is consistent across age groups.

#### 3.3.2 Univariate follow-ups

Univariate analyses focused on the significant differences observed across age categories. Follow-up univariate ANOVAs revealed significant age-related shifts for Alignment of Expectations [*F*(3, 431) = 4.12, *p* = 0.007, $\eta_{p}^{2}$ = 0.026] and Support Network [*F*(3, 431) = 13.28, *p* < 0.001, $\eta_{p}^{2}$ = 0.085]. *Post-hoc* Tukey HSD tests for the Alignment of Expectations dimension showed that U18 athletes perceived significantly lower alignment (*M* = 2.95, *SD* = 0.95) compared to U11 athletes (*M* = 3.48, *SD* = 0.88, *p* = 0.005). Similarly, perceived quality of the Support Network reached a peak in the U11 category (*M* = 4.58, *SD* = 1.05) before declining significantly in the U15 (*M* = 3.73, *SD* = 1.12, *p* < 0.001) and U18 categories (*M* = 3.65, *SD* = 1.08, *p* < 0.001).

### 3.4 Phase 3: predictive modeling of long-term engagement intention

To address the third objective, a multiple linear regression analysis was conducted to determine the relative predictive contribution of the five TDEQ-5 dimensions on athletes’ LTEI, while controlling for age categories and competitive calibers.

#### 3.4.1 Regression assumptions and model fit

Prior to conducting the multiple regression analysis, bivariate correlations were examined to assess the relationships between the five TDEQ-5 dimensions and the Long-Term Engagement Intention (LTEI). As shown in Table 4, all environmental dimensions were significantly and positively correlated with LTEI, with coefficients ranging from *r* = 0.46 to *r* = 0.56 (*p* < 0.001). Preliminary analyses were conducted to ensure that the data met the assumptions for multiple linear regression. The variance inflation factor (VIF) for all predictors ranged from 1.76 to 3.53, well below the common threshold of 5.0, indicating that multicollinearity did not significantly bias the model. Residual analysis confirmed homoscedasticity and the linearity of relationships. The overall regression model with age categories and calibers as control variables was statistically significant, *F*(10, 462) = 18.92, p < 0.001, R^2^ = 0.291 (Adjusted R^2^ = 0.284). This indicates that nearly 30% of the variance in players’ LTEI is explained by the five dimensions of the TDEQ-5.

**Table 4:** Pearson correlations between TDEQ-5 dimensions and LTEI.

Variable	1	2	3	4	5	6
1. HQP	–					
2. LTD	0.77***	–				
3. SN	0.68***	0.65***	–			
4. COM	0.76***	0.78***	0.69***	–		
5. AE	0.59***	0.58***	0.53***	0.61***	–	
6. LTEI	0.51*	0.56*	0.46*	0.49*	0.46*	

HQP, Holistic Quality Preparation; LTD, Long-Term Development; SN, Support Network; COM, Communication; AE, Alignment of Expectations; LTEI, Long-Term Engagement Intentions; *p ≤ 0.05; ***p ≤ 0.001.

#### 3.4.2 Relative impact of environmental dimensions

The results of the multiple regression analysis are presented in Table 5. Among the environmental predictors, Long-Term Development (LTD) emerged as the most substantial correlate (*β* = 0.38, *p* < 0.001), followed by Alignment of Expectations (AE) (β = 0.11, *p* = 0.006). The remaining dimensions—Holistic Quality Preparation, Support Network, and Communication—did not significantly contribute to the model (*p* > 0.05).

**Table 5:** Multiple regression analysis predicting LTEI.

Variable	B	SE	*β*	*t*	*p*	VIF
(Constant)	2.64	0.26		10.23	< 0.001	
Environmental predictors						
LTD	0.37	0.08	0.33	4.82	< 0.001	3.53
AE	0.12	0.04	0.15	2.98	0.003	1.76
HQP	0.04	0.06	0.05	0.71	0.478	3.32
SN	0.05	0.04	0.07	1.20	0.231	2.41
COM	0.05	0.06	0.05	0.71	0.478	3.46
Control variables (calibers)						
A					Ref.	
AA	−0.33	0.09	−0.16	−3.54	< 0.001	1.47
AAA	0.32	0.14	0.11	2.32	0.021	1.65
Control variables (age)						
U11					Ref.	
U13	0.01	0.12	0.01	−0.11	0.913	1.79
U15	−0.24	0.11	0.12	−2.17	0.031	2.11
U18	−0.19	0.14	0.07	−1.41	0.160	1.99

R^2^ = 0.409; Adjusted R^2^ = 0.392; *F*(14, 467) = 21.60; *p* < 0.001; B, Unstandardized coefficient; *β*, Standardized coefficient; SE, Standard Error; VIF, Variance Inflation Factor; HQP, Holistic Quality Preparation; LTD, Long-Term Development; SN, Support Network; COM, Communication; AE, Alignment of Expectations; LTEI, Long-Term Engagement Intentions.

## 4 Discussion

The present investigation aimed to provide a systematic mapping of the talent development environment (TDE) within the Quebec female ice hockey ecosystem. By moving beyond traditional, often reductionist models of talent identification, this study evaluated how the structural and pedagogical components of the environment influence the lived experiences of female athletes. The results offer a nuanced understanding of the developmental climate, highlighting the critical role of LTD and HQP. Furthermore, the findings identify systemic disparities across the talent development pathway, suggesting that while holistic preparation is valued, the perceived sustainability of the developmental journey is the primary driver of persistence for female players.

### 4.1 Psychometric integrity and structural salience

The first objective of this study was to evaluate the structural validity of the TDEQ-5 within the specific sociocultural and linguistic landscape of Quebec female hockey. The Confirmatory Factor Analysis (CFA) results substantiated the original five-factor structure proposed by Martindale et al. (2010), demonstrating that dimensions such as Holistic Quality Preparation (HQP), Long-Term Development (LTD), and Support Network (SN) are conceptually relevant and psychometrically stable within this population. The satisfactory fit indices (CFI > 0.90; RMSEA < 0.08) suggest that the instrument effectively captures the multidimensional nature of the developmental milieu.

However, a critical psychometric observation remains the relatively modest internal consistency observed for the AE dimension. This finding is not an isolated artifact of the current sample, but mirrors persistent challenges documented in international validations (Thomas et al., 2020). Theoretically, the lower reliability of the AE sub-scale may reflect the inherent complexity of the “alignment” construct itself. In the Quebec female hockey pathway, athletes must navigate a convoluted intersection of academic requirements, high-performance training volumes, and varying degrees of family support. Unlike their male counterparts, who often perceive a more standardized professional pathway, female athletes face a more heterogeneous set of expectations regarding their future in the sport (Portela-Pino et al., 2023). This heterogeneity likely introduces greater variance in item responses, suggesting that while “alignment” is an important theoretical pillar, its operationalization through self-report measures remains sensitive to the specific life stages and career uncertainties unique to female sport. Beyond the psychometric validation of the instrument and the LTEI scale, the present findings also provide important insights into how the perceived quality of the development environment varies across competitive levels and age categories within the Quebec female hockey pathway.

### 4.2 The developmental environment and trajectory

A pivotal finding of the present study lies in the surprising homogeneity of environmental perceptions across competitive calibers. Contrary to the popular thought that elite tiers (AAA, AA) are expected to provide superior resources compared to recreational tiers (Ruest, 2020)—our multivariate results revealed no significant differences based on competitive level. This structural homogeneity suggests a successful democratization of developmental standards within the Quebec female hockey ecosystem. Dimensions such as Holistic Quality Preparation (HQP) and Communication (COM) appear to be consistently implemented regardless of the athlete’s competitive standing, reflecting a systemic alignment with the pedagogical mandates of Hockey Québec (2023). From a Holistic Ecological Approach (HEA) perspective, this indicates that the “macro-systemic” standards are well-integrated across different “micro-systems” (teams), providing a stable baseline of technical and organizational support for all female players (Henriksen et al., 2020). This finding is particularly notable when compared to international contexts, such as youth soccer in Western Australia, where significant disparities in perceived environmental quality have been documented between different levels of play and between genders (Lyons et al., 2024). When compared to the Chinese TDE, the Quebec ice hockey TDE.

However, this structural stability is contrasted by a significant developmental friction emerging across the age continuum. While the caliber of play does not differentiate the athletes’ experience, their developmental stage does. The most critical erosion occurs within the Support Network (SN) and Alignment of Expectations (AE) dimensions, particularly as athletes transition into the U15 and U18 categories. The observed decline in perceived support highlights a rupture in the athlete’s social scaffolding, which occurs at a time when the highest rates of sports dropout are reported in the scientific literature (Eime et al., 2019). The literature consistently identifies the quality of the support network and a focus on long-term development as critical predictors of athlete motivation and psychological well-being (Li et al., 2018). Consequently, the erosion of these dimensions may not only jeopardize performance but also increase the risk of athlete burnout, a phenomenon negatively correlated with high-quality talent development environments (Ivarsson et al., 2015). Lack of support, especially from coaches, is also often cited as a primary cause for sports dropout (Back et al., 2022). As players progress toward higher performance demands, the holistic nature of the support may be overshadowed by a narrower focus on technical output, a phenomenon previously noted by Martindale et al. (2010) as a potential risk for long-term persistence. This erosion is most acute in the Alignment of Expectations (AE), which reached its lowest point in the U18 category. A similar trend can be observed in European soccer, where the AE also appears to be declining as players enter the U18 category (Gesbert et al., 2021). This significant drop compared to U11 athletes underscores a growing disconnect between the athlete’s personal aspirations and the systemic pathways available. In the Quebec context, the U18 stage represents a critical juncture at which female athletes must navigate the transition to collegiate (RSEQ) or university hockey (outside the province), often amid significant career uncertainty (Mac, 2022). Unlike male pathways, which are characterized by more standardized professional prospects, female athletes face a “heterogeneity of expectations” (Portela-Pino et al., 2023) that may complicate the perceived coherence of their environment. The decline in AE and SN scores suggests that the environment’s ability to provide a clear, supportive, and aligned vision is diminishing.

When situating these results within the broader international TDEQ literature, the Quebec female hockey environment exhibits a distinct profile. Our total sample reported higher Holistic Quality Preparation (M = 4.55) and Support Network (M = 4.11) scores compared to talented Chinese athletes (M = 3.98 and 4.03, respectively; Li et al., 2018) and Caribbean track and field participants (M = 4.19 and 3.78, respectively; Thomas et al., 2020). These differences likely reflect the advanced integration of resources within Quebec’s Sport-Études programs, which prioritize synchronizing athletic and academic support structures. Another notable cross-cultural divergence was observed in the Communication dimension, where Quebec athletes scored lower (M = 4.24) than their Chinese (M = 4.61) and Caribbean (M = 4.70) counterparts. This disparity may be attributed to the inherent complexity of communication in team sports, compared with the more individualized pedagogical exchanges typical of individual sports or residency-based sports school models. Despite these differences, the observed erosion of Support Networks as athletes progress from U11 (M = 4.58) to U18 (M = 4.10) appears to be a universal developmental phenomenon, consistent with findings across diverse international contexts (Li et al., 2018; Thomas et al., 2020).

### 4.3 Determinants of long-term engagement intentions

The third objective sought to quantify the relative predictive contribution of the TDEQ-5 dimensions on athletes’ LTEI. The regression model highlighted LTD as the primary predictor, followed by AE. This hierarchy confirms that for Quebec’s female hockey players, the intention to persist is fundamentally governed by the perceived clarity and sustainability of their developmental trajectories rather than immediate support structures. This finding is consistent with recent exploratory analyses conducted at international elite academies, in which a long-term development focus was identified as the most stable predictor of athlete commitment (Murray et al., 2027). This finding aligns with the Long-Term Athlete Development framework, which posits that environments prioritizing age-appropriate, logical progression over immediate performance outcomes are superior at fostering long-term athletic commitment (Balyi and Hamilton, 2004; Ford et al., 2011). The fact that LTD and AE emerged as the strongest predictors in Quebec—despite being the dimensions with the lowest mean scores among older athletes—reinforces a broader trend in female hockey.

Similar to the Norwegian context, female athletes’ commitment is fundamentally driven by the perceived sustainability of their trajectory (Mehus et al., 2025). However, the ‘structural friction’ observed in Quebec suggests that the transition to the junior-to-senior level may be more administratively standardized but psychologically more precarious than in systems where individual alignment is prioritized over collective technical homogeneity. Collective technical homogeneity is exemplified by the Quebec ecosystem’s reliance on standardized pedagogical mandates (Hockey Québec, 2023), uniform coaching certifications such as High Performance 1 (Hockey Québec, 2023), and the strictly centralized AAA structure of the LHEQ (LHEQ, 2024). This model ensures technical consistency through formalized programs like Sport-Études (Ministère de l’Éducation, 2022), yet its inherent rigidity contrasts with the ‘individual alignment’ characteristic of decentralized, club-based Nordic systems. In the latter, developmental pathways are more flexibly adapted to the athlete’s unique academic requirements and personal aspirations, fostering a ‘milieu-specific’ rather than ‘system-specific’ support structure (Henriksen et al., 2020; Mehus et al., 2025). In a sport where female professional pathways are still maturing, the athlete’s belief that her current environment is preparing her for future stages of excellence appears to be the most critical factor for retention (Stambulova and Wylleman, 2014; Manaf et al., 2022). This is particularly crucial as research indicates that a focus on long-term goals is strongly associated with higher levels of intrinsic motivation and psychological well-being in youth athletes (Li et al., 2018). This echoes the statements of Lloyd et al. (2016), who argue that pillars of an LTAD approach include promoting young people’s well-being, minimizing injuries, fostering each athlete’s development, and increasing the likelihood that they will choose to remain involved in their sport of choice. The addition of AE as a significant predictor reinforces the Holistic Ecological Approach (HEA) to talent development (Henriksen et al., 2010). According to this perspective, successful development environments do not treat sport in a vacuum but as part of a broader ecosystem where the athlete’s non-sporting life and support structures are integrated. The predictive weight of AE indicates that even if high-quality resources (HQP) are present, their impact on long-term commitment is diminished if there is a disconnect between the system’s expectations and the athlete’s personal goals (Gesbert et al., 2021). Henriksen and Stambulova (2017) emphasize the importance of the HEA for student-athletes and state that educational institutions must make an effort to integrate and prioritize sports, while better equipping athletes to advocate for their various needs.

55 Previous research utilizing the TDEQ has similarly demonstrated that favorable perceptions of the development environment are positively associated with higher levels of motivation, wellbeing, and a reduced risk of burnout (Ivarsson et al., 2015; Wang J. C. et al., 2011; Wang C. K. J. et al., 2011). For female athletes, who often perceive their developmental environments as less supportive or more uncertain than their male counterparts (Lyons et al., 2024), the clarity provided by LTD and AE acts as a vital psychological anchor. This is particularly relevant given the high dropout rates observed among adolescent girls; according to the Canadian Women and Sport Rally Report (2024), nearly 1 in 2 girls leave sport by age 16. Our results suggest that mitigating this attrition requires more than just technical or material support; it necessitates an environment that prioritizes the “whole person” by synchronizing personal aspirations with a transparent and long-term developmental roadmap (Wang et al., 2016).

### 4.4 Practical applications for stakeholders and decision-makers

The findings of this study offer empirical directives for ice hockey stakeholders, regional directors, and coaching staffs, providing a framework to optimize the developmental climate for female athletes. The prominence of LTD as the primary predictor of engagement suggests that athletes’ psychological commitment is fundamentally linked to the clarity and sustainability of their developmental roadmap. Consequently, organizations should formalize coaching strategies that prioritize age-appropriate, long-term progression over immediate performance outcomes. This includes implementing standardized individual development plans that track technical and psychological growth across seasons, ensuring that the “next step” in the pathway is always visible and attainable for the player. By centering the environment on long-term goals, stakeholders can foster higher levels of intrinsic motivation and psychological well-being, which are essential for navigating the demands of competitive sport (Li et al., 2018; Manaf et al., 2022).

Furthermore, the observation of structural homogeneity across competitive calibres indicates that Hockey Quebec’s pedagogical standards are being consistently applied throughout the ecosystem. Stakeholders should prioritize sustaining the quality of the SN, which shows a significant decline as athletes progress into late adolescence. To mitigate this developmental friction, specialized workshops for coaches in the U15 and U18 categories should be developed to address the specific social and emotional needs of adolescent female players (Lyons et al., 2024). Strengthening the coach-athlete relationship during these critical stages can serve as a protective mechanism against the increased risk of burnout and dropout that often emerges when technical demands begin to overshadow holistic support (Back et al., 2022).

Finally, the decline in AE at the U18 level requires targeted institutional interventions to manage the “bottleneck” phase of the junior-to-senior transition. Stakeholders must provide transparent pathway counseling for athletes approaching the end of their minor hockey journey, specifically regarding the transition to collegiate (RSEQ) or university levels. As professional opportunities for women continue to evolve with the PWHL, organizations have a responsibility to align their developmental objectives with these emerging professional realities. Establishing formal mentorship programs where older athletes can interact with collegiate or professional players could reduce the career uncertainty that clouds the U18 experience. Such interventions are crucial for maintaining vocational alignment and supporting athletes during the precarious shift toward higher performance levels (Thomas et al., 2020; Stambulova and Wylleman, 2014).

### 4.5 Limitations and perspectives for future research

Despite the methodological rigor and the relatively large sample size, certain limitations must be acknowledged. First, the cross-sectional nature of the data prevents the establishment of definitive causal relationships between environmental quality and athlete perceptions. While the results demonstrate that the environment is linked with satisfaction, it is also possible that satisfied athletes perceive their milieu more favorably. Future research should employ longitudinal designs to track how perceptions of the TDE evolve across multiple seasons and during critical transitions between recreational, competitive and elite levels.

Second, the reliance on self-report measures introduces potential biases, such as social desirability or the temporary influence of end-of-season emotional states. While the TDEQ-5 is a validated tool, future investigations would benefit from a multi-informant approach, incorporating the perspectives of parents and coaches to provide a complete view of the developmental climate. Additionally, the modest reliability of the AE dimension warrants a qualitative investigation. This low consistency, frequently observed in TDEQ-5 validations, may stem from specific item—such as Item 9 —which often yield weak factor loadings across different athletic populations (Thomas et al., 2020). Semi-structured interviews with U18 players could reveal the specific socio-structural tensions that contribute to the perceived misalignment of expectations, offering a depth of insight that quantitative scales may fail to capture.

Another methodological limitation of this research resides in the inherent selection or “survivor” bias of the sampling procedure. As only athletes actively engaged in hockey at the time of the investigation were solicited, the perceptions of players who had already decided to withdraw from the sport remain unknown. Furthermore, only one team per calibre, age and region were sent to this end-of-season tournament, skewing the results toward successful teams. The omission of these perspectives restricts our understanding of the specific environmental variables that may have precipitated athletic disengagement prior to data collection. This finding reinforces the critical need for longitudinal research designs that enable tracking of athletes’ trajectories in real time, as well as retrospective studies that specifically address the views of former players. Such an approach would allow for a comparative analysis of perceptions between persistent athletes and those who have discontinued, thereby offering a more comprehensive and systemic understanding of retention and attrition factors within female ice hockey.

## 5 Conclusion

This study provides the first empirical mapping of the Quebec female hockey environment. While the system appears to offer structural equity across calibers, the findings highlight potential attrition factors related to the erosion of social support and challenges in vocational alignment during late adolescence. These results suggest that sustaining long-term commitment may require going beyond technical instruction to incorporate a more holistic, athlete-centered approach that considers the broader developmental context. Although not directly examined in the present study, these findings may have implications for how development systems align with the evolving professional landscape of women’s hockey. In particular, fostering clearer connections between developmental environments and emerging professional opportunities could represent a promising avenue to support athlete retention during critical transition periods. Situated within the TDEQ and Talent Development literature, this study underscores both the potential benefits and limitations of a standardized and centralized development structure, particularly in relation to supporting the long-term engagement of female athletes.

## Data Availability

The raw data supporting the conclusions of this article will be made available by the authors, without undue reservation.
